# Supplementary material for: Structures of the archaerhodopsin-3 transporter reveal that disordering of internal water networks underpins receptor sensitization
Source: Nat Commun. 2021 Jan 27;12:629. doi: 10.1038/s41467-020-20596-0 (PMC7840839; doi:10.1038/s41467-020-20596-0)
Supplement: Supplementary file 1 — Supplementary Information [file 41467_2020_20596_MOESM1_ESM.pdf]

Structures of the archaerhodopsin-3 transporter reveal that disordering of internal water networks underpins receptor sensitization

Supplementary Information

Methods

Protein expression

Halorubrum sodomense (ATCC-33755) cells were purchased from LGC Standards Ltd (Teddington, UK) and were grown without any genetic modification. Initially cells were grown in 4x 25 ml of liquid culture media at pH 7.4 with high salt concentration (per litre: 125 g NaCl, 160 g $MgCl_2$, 0.13 g $CaCl_2$, 5 g K_2SO_4, 1g bacteriological peptone (Oxoid, UK), 1 g yeast extract (Melford, UK), 2 g soluble starch (Sigma)) at 45 °C and 170 rpm shaking for 5-7 days, or until the OD_{600} reached 1.2. Fresh culture medium (of identical composition) was inoculated from the 25 ml cultures and incubated at 45 °C and 170 rpm shaking for approximately three weeks until reaching an OD_{600} of 1.9.

Protein delipidation

Native claret membrane vesicles were pelleted by centrifugation (70,000*g*, 30 min, 4 °C) and resuspended in 6 ml of 25 mM NaH_2PO_4, pH 6.9, and 2 ml of 10% OG. The preparation was sonicated for 1 min at room temperature in a bath sonicator and incubated overnight at 22 °C without stirring. The solution was adjusted to pH 5.5, and centrifuged (100,000*g*, 45 min, 15 °C) to remove aggregated and non-solubilized material. Finally, the sample was applied to a preparative gel column (Hi-Load 16/600, Superdex 200 pg) pre-equilibrated with 1.2% n-octyl-β-D-glucoside (OG) (Anatrace, Maumee OH, USA) in 25 mM NaH_2PO_4, pH 5.5[1]. The colored fractions were pooled and concentrated up to 9 mg/ml.

Spectroscopy

UV/Vis absorption spectra were acquired using a Jasco V-630 UV spectrometer and all samples were blanked against buffer solutions. The Synchrotron Radiation Circular Dichroism (SRCD) spectra in the far-UV region were collected at the Diamond B23 beamline module end station B (Diamond Light Source Ltd., UK) using a demountable quartz cell of 0.02 cm path length (Suprasil cell, Hellma Analytics, Müllheim, Germany). A wavelength range (185-240 nm) was selected to monitor secondary structure of the proteins at ambient temperature. Protein concentration was in the range of 0.5-1 mg/ml in low salt buffer in order to perform experiments. The SRCD spectra were processed and baseline corrected using the CDApps software[2]. Secondary structure content was calculated from the Dichroweb server[3] using the CDSSTR analysis programme[4] and the reference set SMP180 for membrane proteins[5]. Spectra were prepared for publication using Prism version 8 (GraphPad, CA, USA).

Native mass spectrometry

Purified AR3 was buffered exchanged into 0.2 M ammonium acetate containing a mixture of *n*-Dodecyl-β-D-maltoside (DDM), foscholine and cholesteryl hemisuccinate[6]. The sample suspension was immediately introduced into a prototype Q Exactive plus hybrid quadrupole-Orbitrap mass spectrometer[7–9] through nano-electrospray (nESI) at capillary voltage 1.4 kV. Protein complexes were liberated from detergent micelles in the injection flatapole at 100 V in-source trapping (IST) voltage. Ion optics were optimized to transmit ions with minimal activation (inter-flatapole, bent flatapole, and transfer multipole: 6.94 V, 5.9 V, and 4 V, respectively). For MS/MS experiments[10], protein-lipid

complexes were isolated by the quadrupole with 5 m/z isolation window width and collisional activation (0 V—100 V, argon UHV pressure 10-9 mbar) was applied in the HCD cell.

Atomic Force Microscopy

Membranes were coated on mica in buffer solution (Tris 20 mM, 150 mM KCl, 10 mM $MgCl_2$, pH 7.4) for 15 min and the topograph was recorded in the same buffer but containing 300 mM KCl and no divalent cations. Images were recorded in tapping mode with MSNL cantilevers, using a Multimode equipped with a Nanoscope 3a controller and an E scanner (NanoBruker, Germany).

Molecular dynamics simulations

For the simulation, standard protonation states were chosen for all amino acid residues of AR3 except for Glu214, which was modelled as protonated. Using CHARMM-GUI[11–15], the protein was then embedded in a hydrated 1-palmitoyl-2-oleoyl-*sn*-glycero-3-phosphocholine (POPC) membrane of dimensions 90×90×100 $Å^3$ (~75,000 atoms in total). Seven sodium ions were added to the setup to ensure overall charge neutrality. The AMBER16 software package[16] was employed to run two independent MD simulations for each of the retinal conformations found in the two crystal structures. A Langevin dynamics scheme was used with the CHARMM36 protein[17] and lipid force field[18], the TIP3P water model[19] and the ion parameters by Roux and coworkers[20]. Hydrogen-containing bonds were constrained with the SHAKE algorithm[21]. Each setup was equilibrated for 30 ns. Restraints were placed on the heavy atoms and they were gradually released throughout the equilibration phase. During the initial 750 ps of equilibration the system was heated from 100 K to 300 K as a canonical ensemble (NVT). Afterwards an isothermal–isobaric ensemble (NPT) was employed at a pressure of 1 bar. Finally, 300 ns production runs were initiated, and the integration time step was increased from 1 fs to 2 fs. Coordinates of all atoms were saved in 10 ps intervals. Water densities were generated with the VolMap plugin in VMD (version 1.9.3)[22] by averaging the occupancy of the water oxygens for the whole simulation. The water densities were visualized with PyMOL (versions 2.2 and 2.3.0)[23].

Results

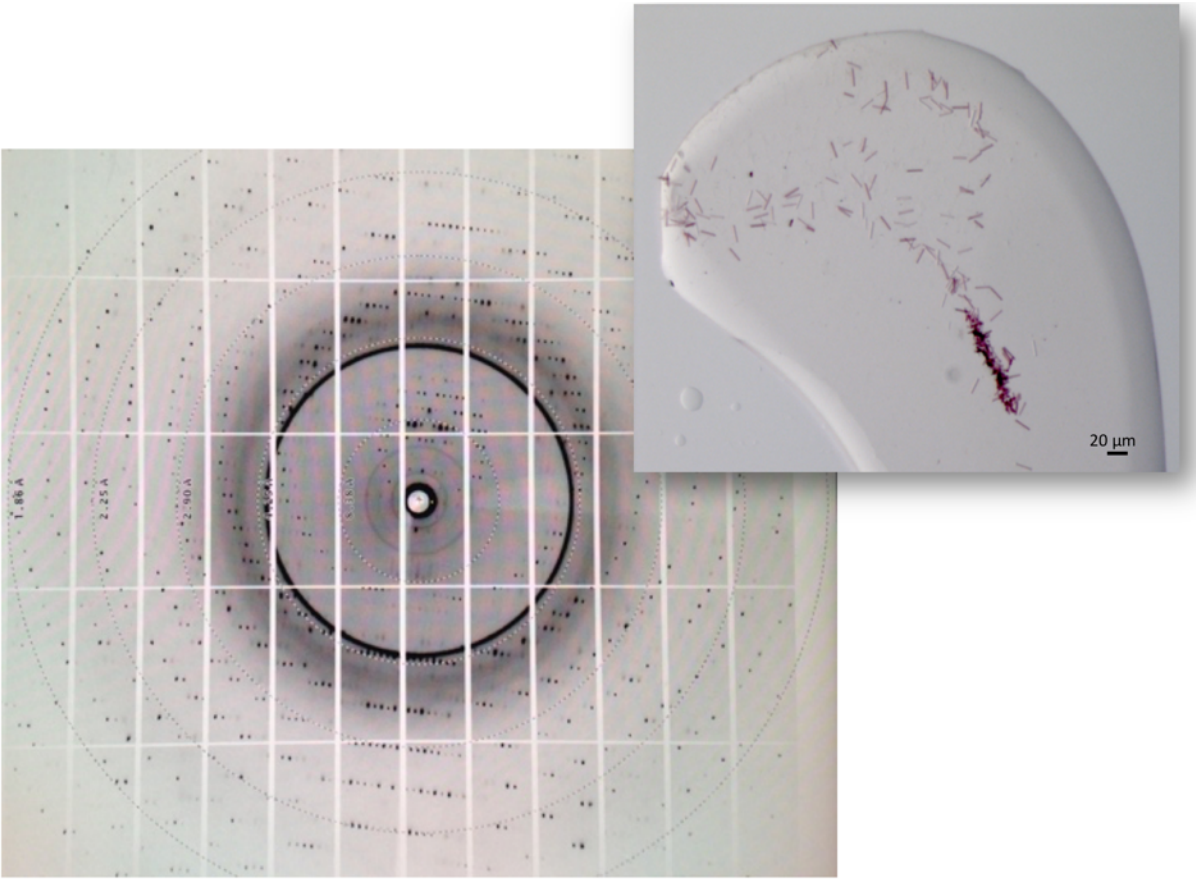

Supplementary Figure 1| Typical AR3 crystals and representative diffraction data. Crystals of Archaerhodopsin-3 (AR3) were grown in lipidic cubic phase **(inset)** and a representative X-ray diffraction pattern for AR3 crystals under cryo-conditions **(main)** when exposed at I24 microfocus beamline, Diamond Light Source, UK. Crystals were grown reproducibly from three separate protein preparations. In each case X-ray diffraction patterns of similarly high resolution were obtained.

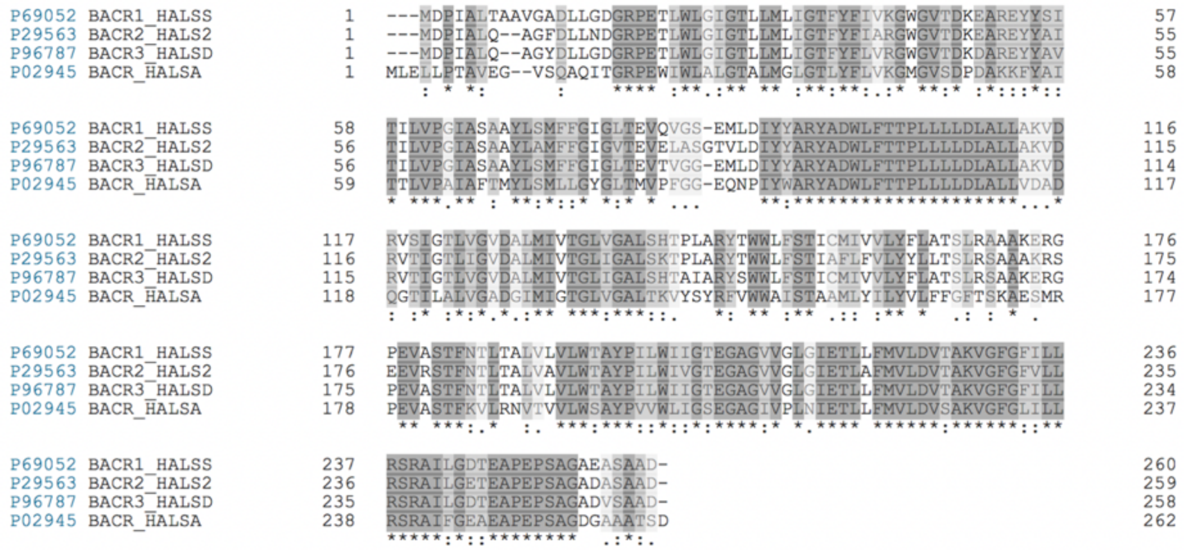

Supplementary Figure 2| Sequence comparison of AR3 and related microbial rhodopsins. The amino acid sequences of AR1 (BACR1_HALSS), AR2 (BACR2_HALS2), AR3 (BACR3_HALSD), and bR (BACR_HALSA), as translated from the gene sequences deposited in the UniProt database are shown. Identical residues are highlighted in dark grey, similar residues are highlighted in light grey. Note that this sequence alignment does not indicate post-translational modifications.

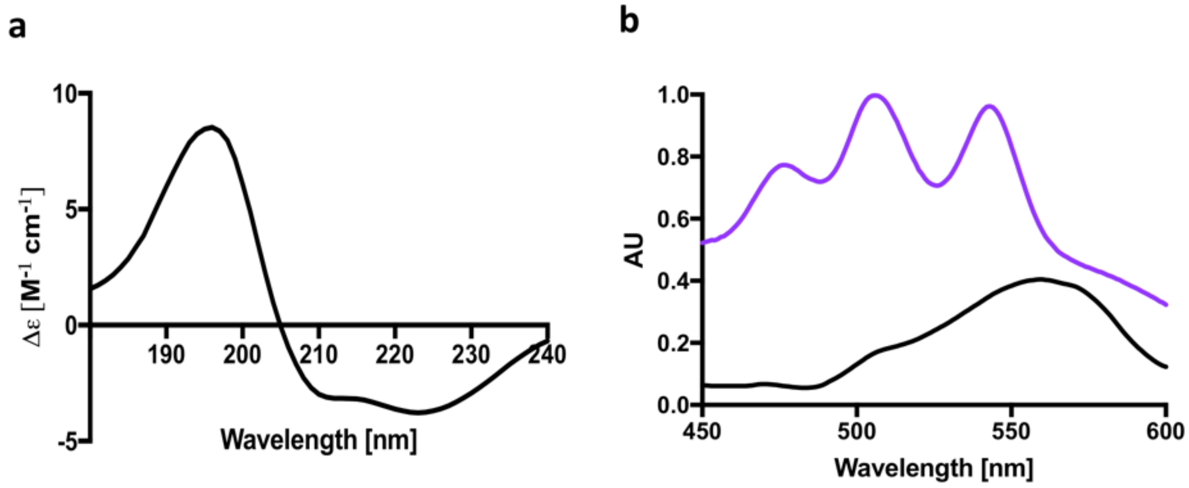

Supplementary Figure 3| Circular dichroism (CD) and absorption spectra of AR3. The synchrotron CD spectrum of AR3 in claret membrane vesicles **(a)** was recorded on the B23 beamline at Diamond Light Source. Analysis of the CD spectrum using the CDSSTR[3] algorithm predicts an alpha-helical content of 74%**.** Absorption spectra of AR3 in vesicles of the claret membrane (purple) and delipidated with OG to remove the bacterioruberin chromophore (black), indicate that the mature AR3 protein has a maximum absorbance of 555 nm **(b)**. It was not possible to remove all the bacterioruberin from the sample, as evidenced by the shoulder visible in the spectrum around 510 nm. The absorption spectra were acquired on a Jasco V-630 UV-Vis spectrometer.

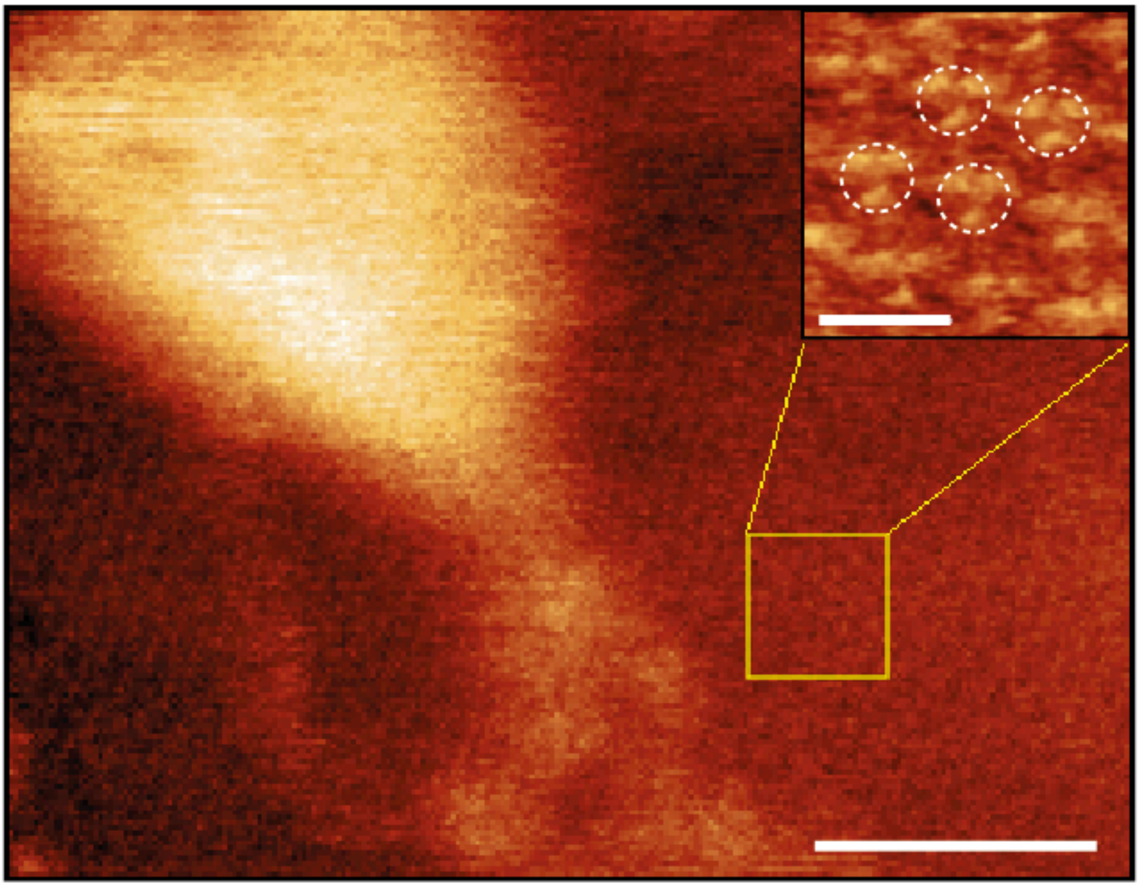

Supplementary Figure 4| Atomic Force Microscope (AFM) images of AR3. AFM height image of a 200 nm scan of an AR3 2D crystalline array in the claret membrane (main picture). The lattice could be successfully imaged at this magnification. The inset shows the AR3 trimeric organisation (trimers are indicated by the white dashed circles). The yellow square indicates the imaged area shown in the inset. The scale bars are 50 nm (main image) and 10 nm (for the inset). AR3 lattice dimensions for this image are indicated in Supplementary Table 2. Three independent samples were imaged and the results were found to be reproducible.

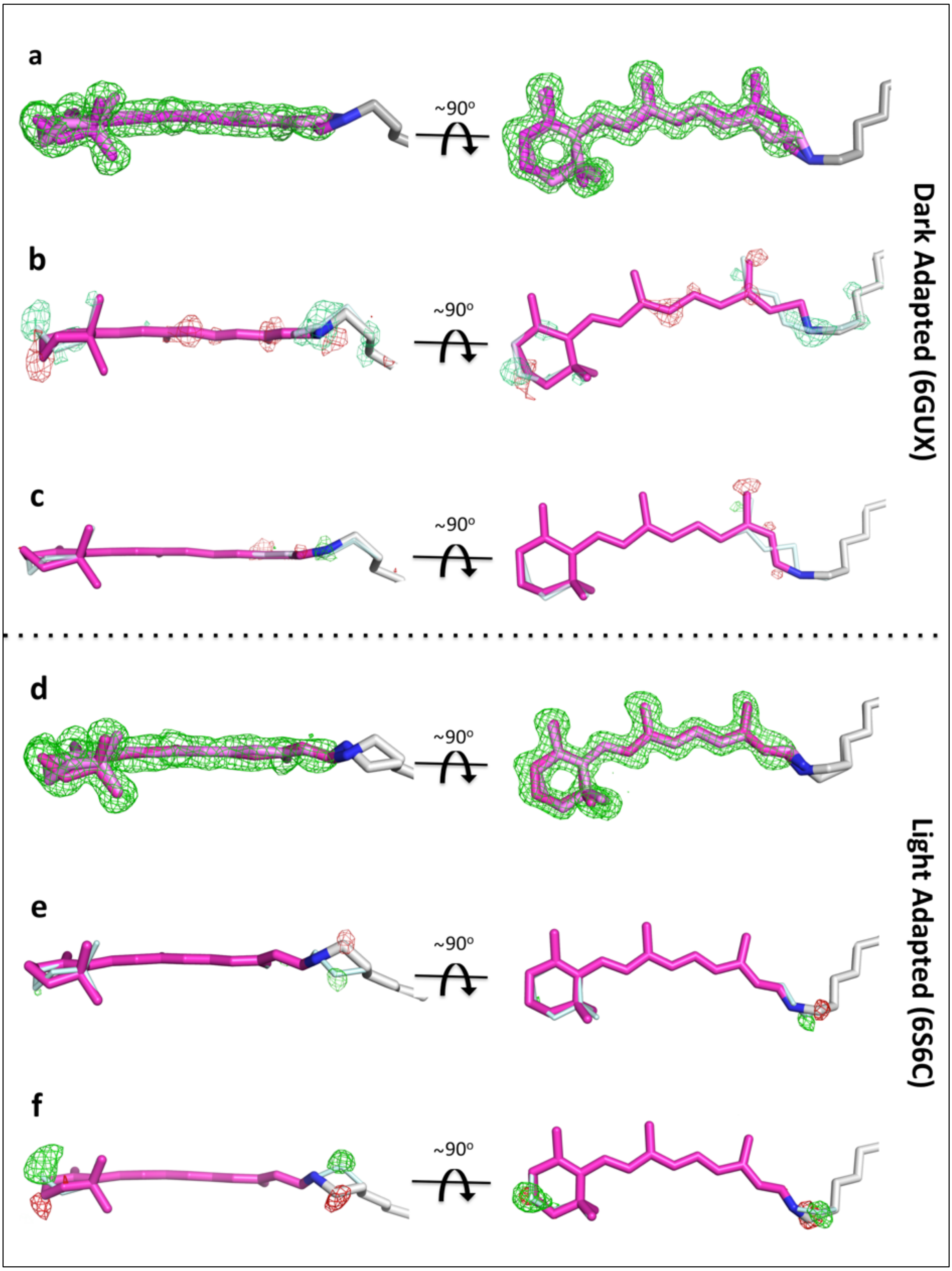

Supplementary Figure 5| Validation of the retinal and SB lysine conformations by omit electron density maps (mF_{obs}-DF_{calc}). Panels on the left represent the bottom view of the retinal while panels on the right represent the retinal side view. **a, d** Strong positive density (green mesh) in the shape of retinal is observed when retinal conformations (represented here in dark and light magenta sticks) are totally omitted during refinement (mF_{obs}-DF_{calc},

±3σ). **b, c, e, f** Fourier electron density maps (mF_{obs}-DF_{calc} contoured at ±3σ) around the retinal and Lys226 when one of the conformations is removed. The resultant strong positive (green mesh) and negative (red mesh) peaks clearly indicate the presence of the retinal and Schiff base lysine in multiple conformations in both DA and LA structures. Bold sticks represent retinal (magenta) and Lys226 (white) conformers in 100% occupancy respectively, the SB N atom is colored blue. Thin sticks in light cyan represent the omitted conformation.

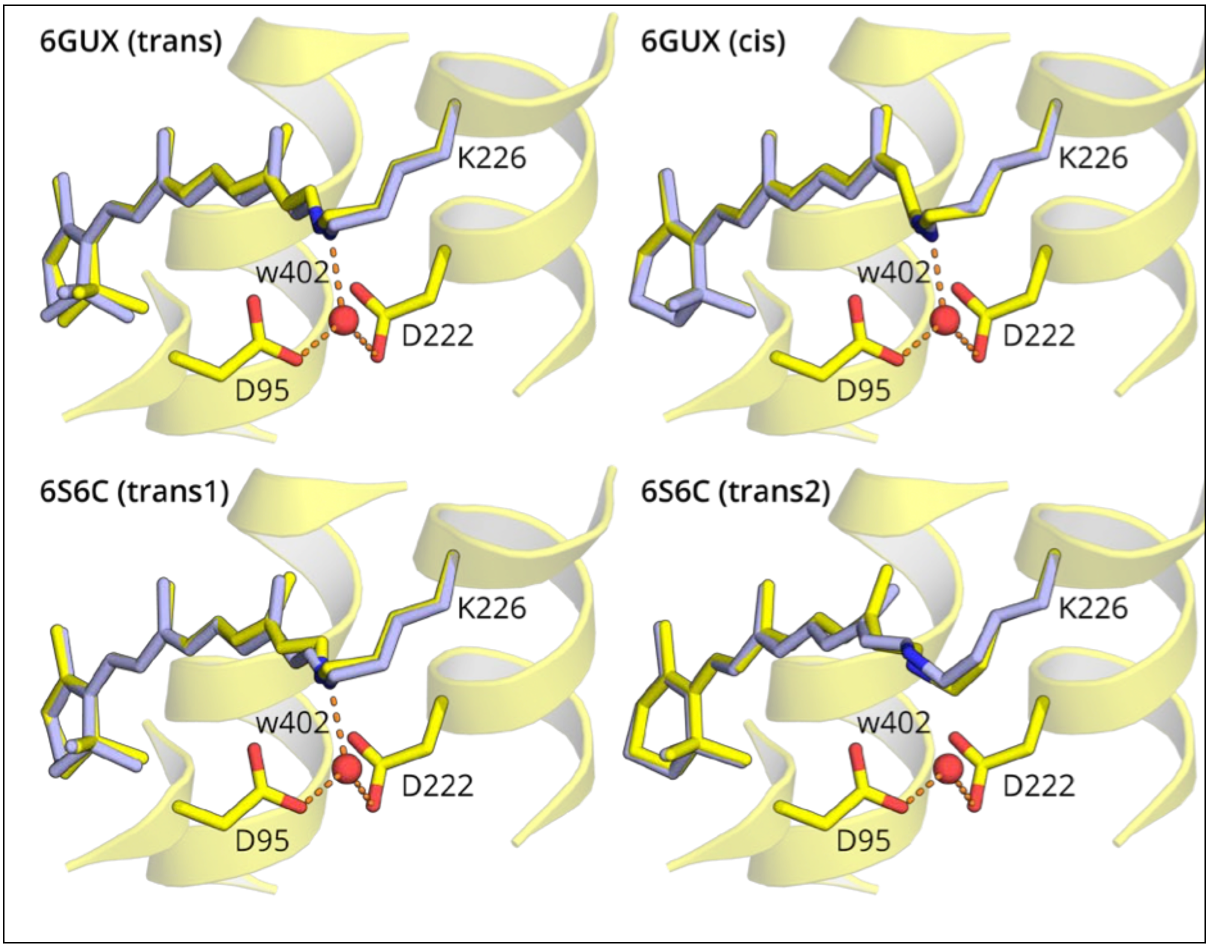

Supplementary Figure 6| QM/MM-optimized structures of the retinal protonated Schiff base, generated using the 6GUX and 6S6C crystal structures as a starting point. We optimized the geometry of the retinal and Lys226 using a QM approach. Only the RPSB was allowed to relax during the optimisation and all other atoms were fixed. The optimized geometry is shown in light blue and the atom positions from the crystal structures in yellow. Water molecules are shown as red spheres and predicted H bonds as dashed red lines. For the 13-*cis* isomer of the dark-adapted form, the crystal and optimized structures are almost identical. The optimized all-*trans* isomer, however, deviates slightly from the crystal, particularly at the Schiff base. Light-adapted AR3 also has the largest deviations close to the Schiff base.

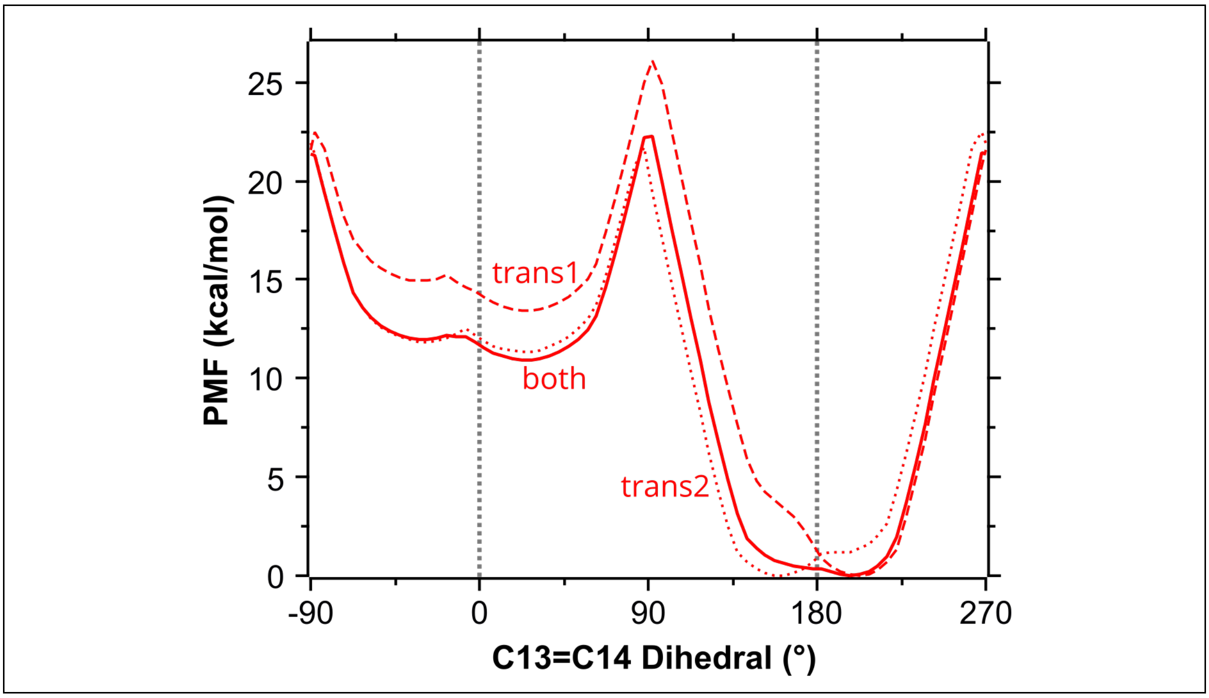

Supplementary Figure 7| Calculated potentials of mean force (PMF) for the isomerisation of the C12-C13=C14-C15 dihedral of retinal for the 6S6C structure. The PMF was computed by sampling the retinal isomerization from all-*trans* to 13-*cis*. Each point on the curve is generated from two independent 0.5 ns QM(SCC-DFTB)/MM MD trajectories, initiated from two separated equilibrated starting structures. The protein backbone was fixed in place, however all other atoms (including the retinal chromophore and amino acid sidechains) were allowed to move. The profile labelled 'both' (solid line) shows the PMF derived from both the trans1 and trans2 states; whereas 'trans1' (dashed line) and 'trans2' (dotted line) represent PMF profiles derived only from the trans1 or trans2 state, respectively.

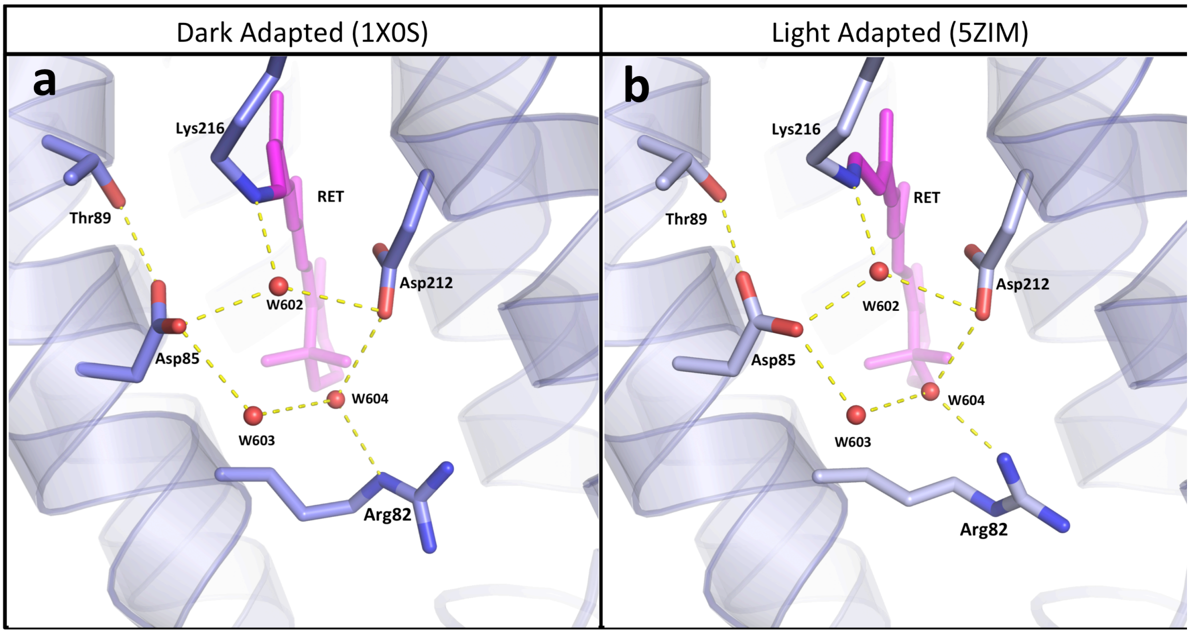

Supplementary Figure 8| Pentagonal H-bond networks in the dark-adapted (DA) state (PDB:1X0S, **a**) and light-adapted (LA) state of bR (PDB:5ZIM, **b**). The hydrogen bond between W602 and the SB Nζ atom is stretched upon retinal isomerization in bR, allowing W602 to become disordered[24,25]. Selected amino acids sidechains are shown in sticks representation with atoms colored using the CPK convention. Water molecules are shown as red spheres and retinal is colored pink. Predicted hydrogen bonds are shown as dashed yellow lines.

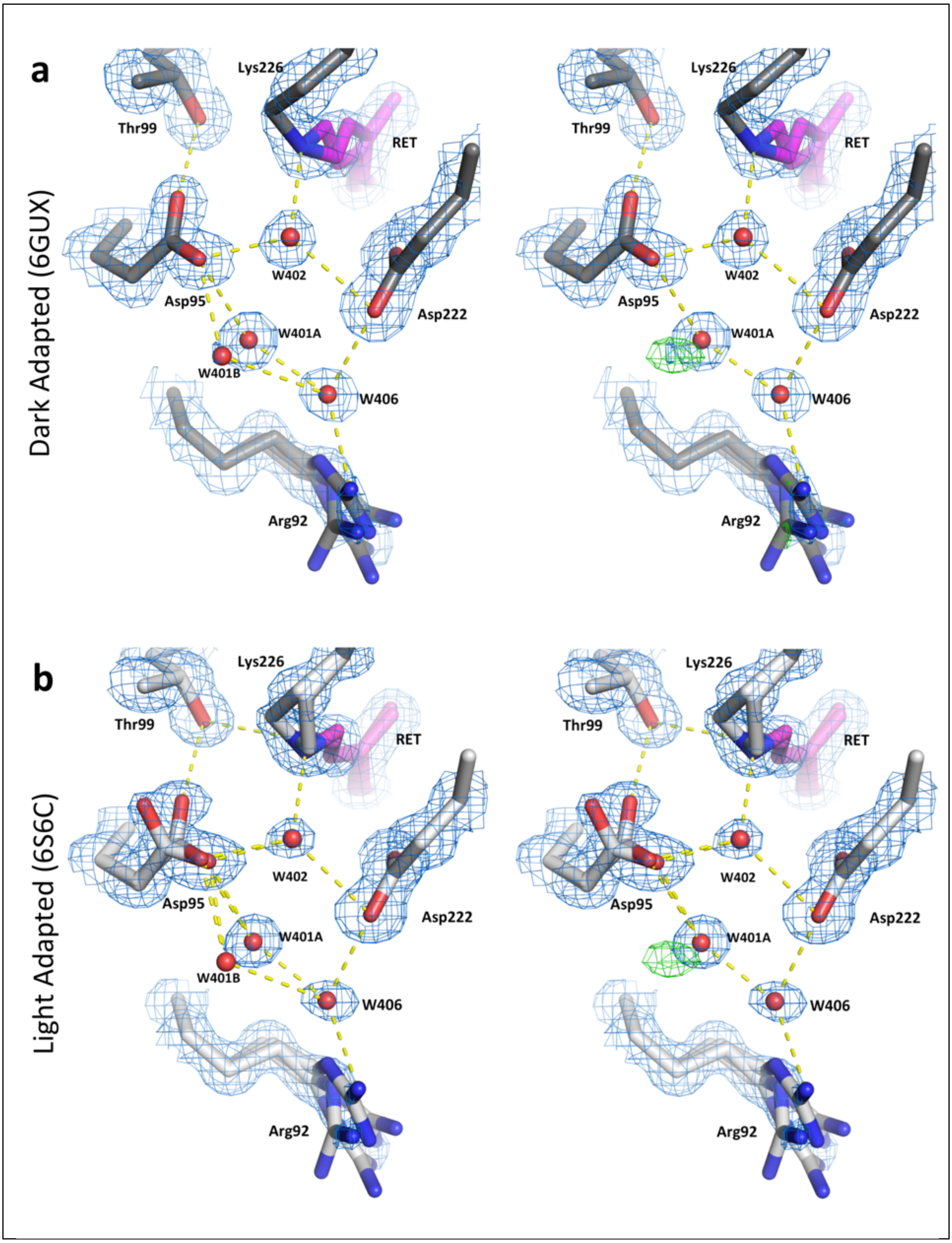

Supplementary Figure 9| Structures of the region between the retinal SB and Arg92 for (**a**) dark-adapted and (**b**) light-adapted AR3. The mF_{obs}-DF_{calc} electron density map (blue mesh) is contoured at ±2.3σ. The right-hand panels for each state, show positive density (green mesh) when the B position of Water401 is omitted during refinement (mF_{obs}-DF_{calc}, ±3σ). Selected amino acids sidechains are shown in sticks representation with atoms colored using the CPK convention. Water molecules are shown as red spheres and retinal chromophore is colored pink. Predicted hydrogen bonds are shown as dashed yellow lines.

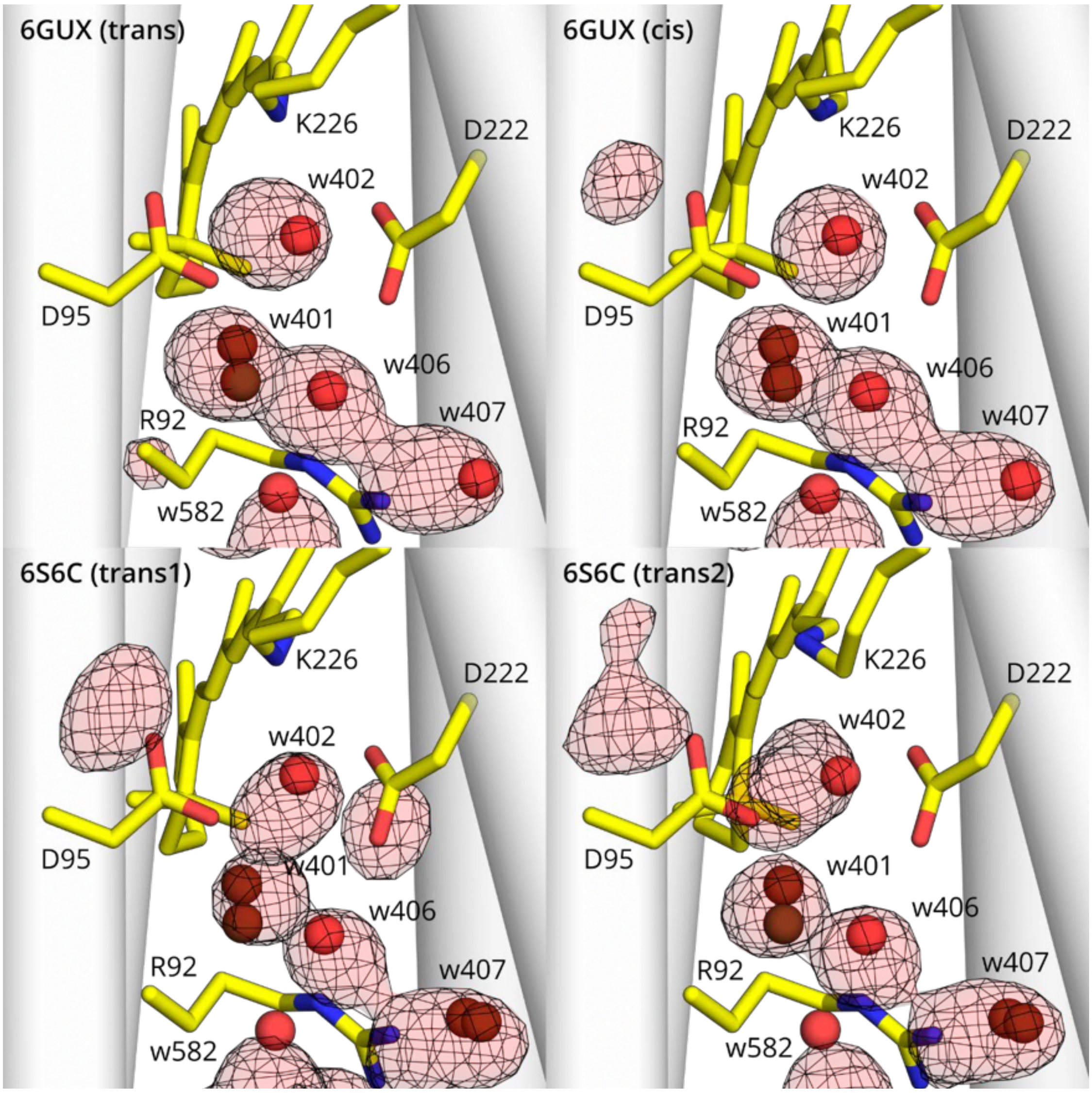

Supplementary Figure 10 | Water density inside DA and LA AR3 during classical MD simulations. We measured the water density in the active site during our MD simulations (light red mesh) and compared it to the positions of the waters from the crystal structures (solid red spheres). All of the crystal water positions were hydrated during our simulations. Our simulations predict an additional water close to D95 for light-adapted AR3 and possibly for dark-adapted AR3 with 13-*cis* retinal. Selected amino acids sidechains are shown in sticks representation with atoms colored using the CPK convention. Water molecules are shown as red spheres and retinal is colored pink.

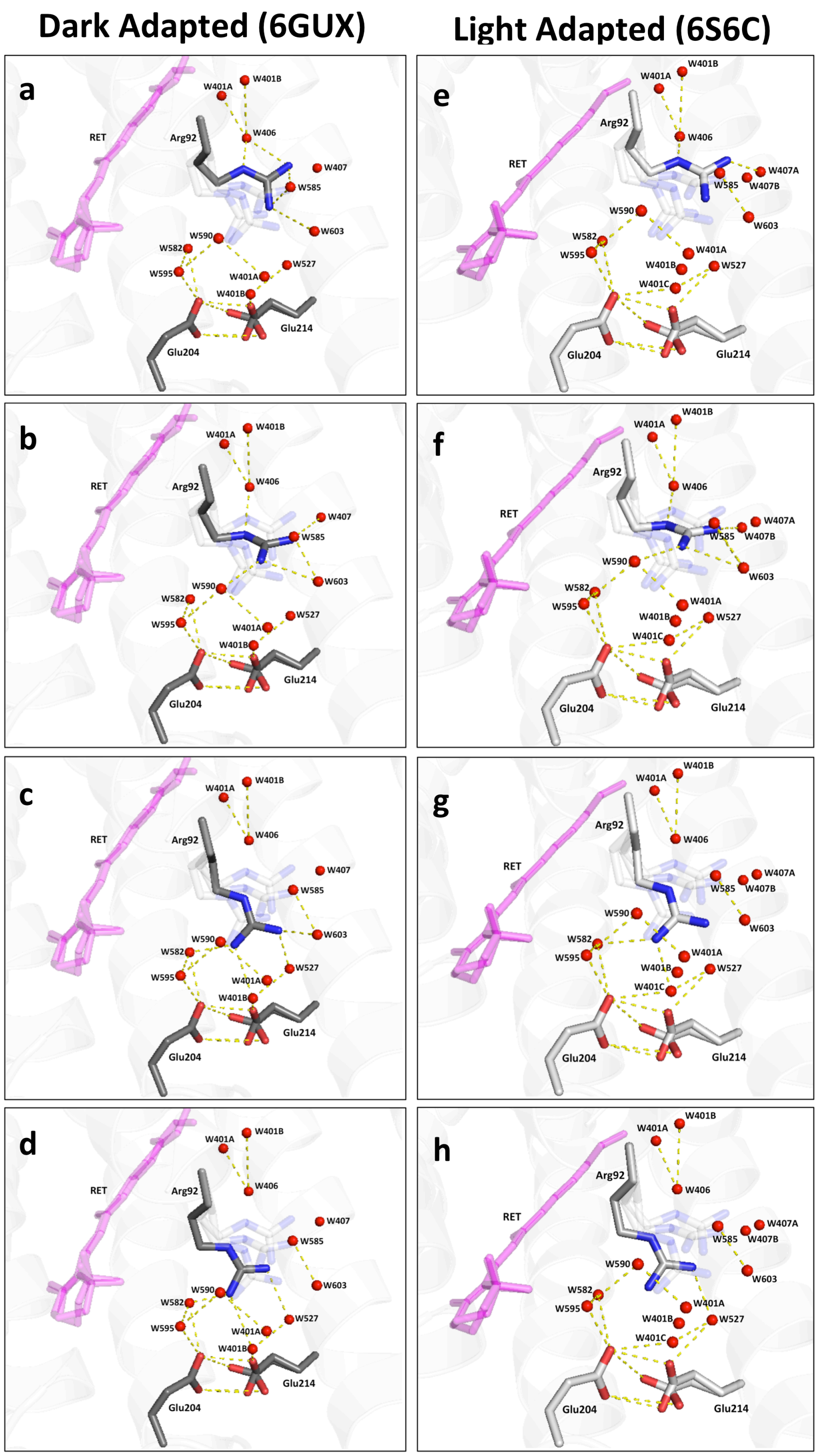

Supplementary Figure 11 | H-bond networks between Arg92 and the Proton Release Complex (Glu204, Glu214) stabilized by different conformations of Arg92 in the DA (**a**, **b**, **c**, **d**) and LA (**e**, **f**, **g**, **h**) states of AR3. One conformation of Arg92 is selected per panel. The yellow dashed lines indicate predicted hydrogen bonds. Selected amino acids sidechains are shown in sticks representation with atoms colored using the CPK convention. Water

molecules are shown as red spheres and retinal is colored pink. Predicted hydrogen bonds are shown as dashed yellow lines.

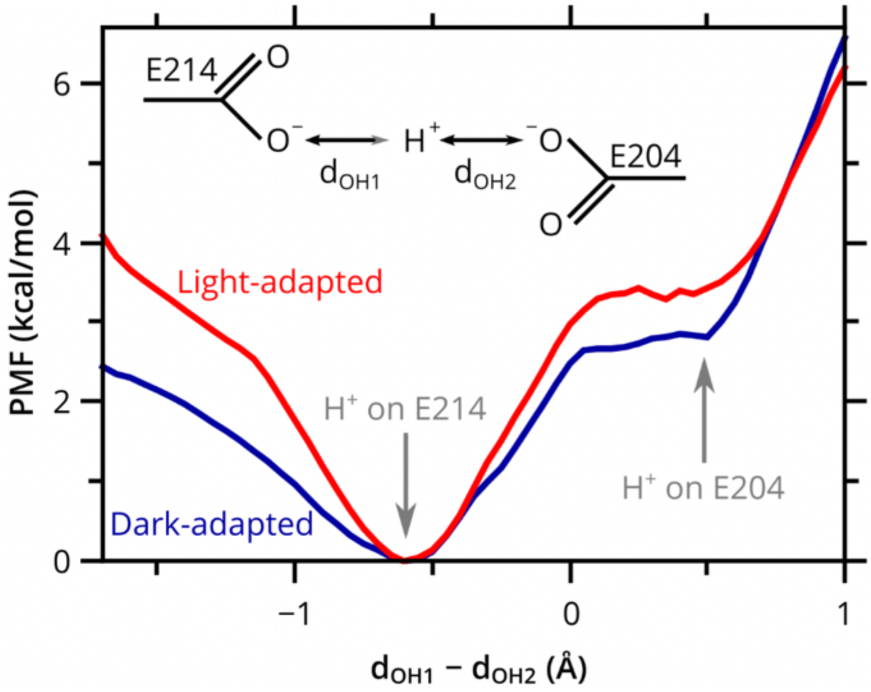

Supplementary Figure 12 | Calculated PMF for proton sharing between E214 and E204 at the extracellular face of AR3. We computed the PMF profile for proton transfer from E214 to E204 and vice-versa. As reaction coordinate, we took the difference between the proton–E214 distance (d_{OH1}) and the proton–E204 distance (d_{OH2}). The resulting graph shows that for both light- (red) and dark-adapted AR3 (blue) protonated E214 is the preferred state.

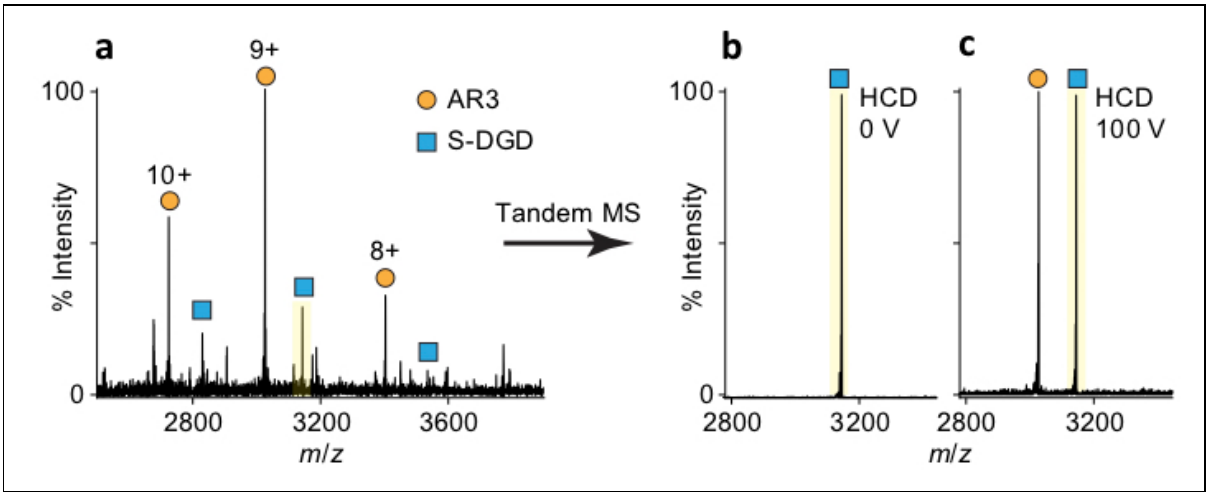

Supplementary Figure 13 | Native mass spectroscopy of AR3 solubilized in detergent (OG). Nano electrospray ionisation spectrum **(a)** showing peaks corresponding to the mature AR3 protein (indicated by orange circles) with a measured mass of 27238 Da (contrasting with a predicted mass of 27746 Da (REF-SWISSPROT BACR3_HALSD) for the monomeric, unmodified AR3 precursor protein). A complex (blue squares) between AR3 and an archaeal lipid (S-DGD) is also detected, with a measured mass of 28456 Da. Tandem MS after selection of the AR3-S-DGD complex at 0 V **(b)** and 100 V **(c)**. Partial dissociation of the complex is observed at an excitation voltage of 100 V, indicating that the lipid is not covalently bound to the protein.

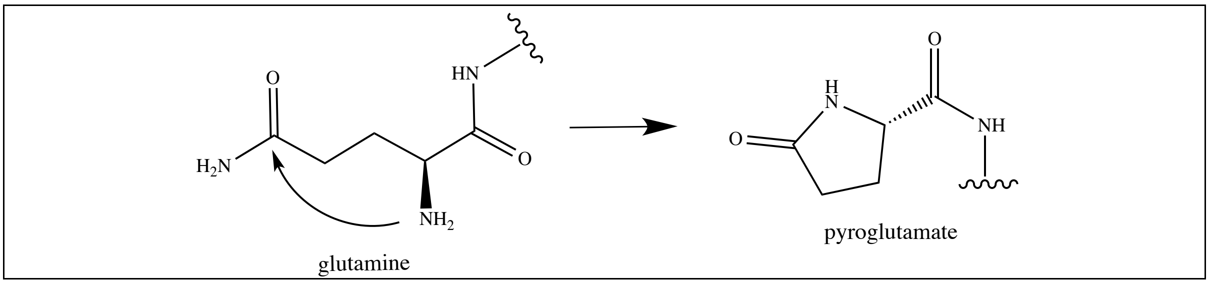

Supplementary Figure 14 | Gln7 is modified to form a cyclic pyroglutamyl residue, which eliminates the primary amine group at the N-terminus of the mature AR3 protein.

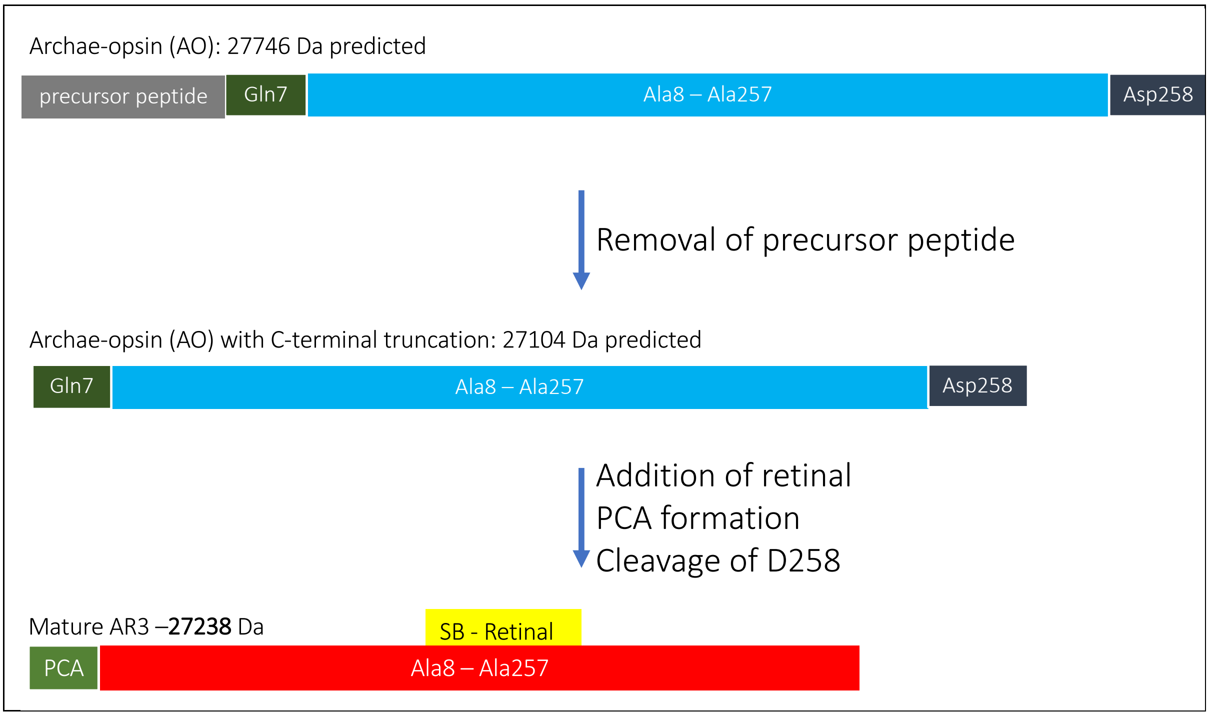

Supplementary Figure 15 | Covalent modification of the AR3 precursor protein (above, MW 27746 Da) to form the mature AR3 protein (below, MW 27238 Da), based on the masses observed by MS (Supplementary Figure 13). The signal peptide (the first six amino acids at the N-terminus) and Asp258 (the final amino acid at the C-terminus) are permanently cleaved. Residue Gln7 is modified to become a pyroglutamyl group eliminating the primary amine group in this position and retinal is covalently conjugated to Lys226 via a Schiff Base.

Supplementary Table 1

	Dark-adapted (DA) structure of Archaerhodopsin-3 (6GUX)	Light-adapted (LA) ground state structure of Archaerhodopsin-3 (6S6C)
**Data Collection**		
Space group	$P2_12_12_1$	$P2_12_12_1$
Temperature	100 K	100 K
Number of crystals	2	17
Cell dimensions		
a, b, c (Å)	46.70, 47.52, 104.42	44.67, 47.36, 104.19
α, β, γ, (°)	90, 90, 90	90, 90, 90
Wavelength (Å)	0.96862	0.96862
Resolution range (Å)	44.74 – 1.20 (1.22-1.20)	52.10 – 1.07 (1.09-1.07)
No. of unique obs.	66292 (2345)	98595 (4610)
Completeness (%)	94.5 (68.8)	99.8 (94.8)
Redundancy	8.20 (2.10)	22.8 (15.0)
R_{pim}@	0.089 (3.253)	0.044 (1.717)
R_{meas}+	0.201 (4.970)	0.208 (6.814)
$CC_{1/2}$	0.994 (0.057)	0.999 (0.179)
Mean $I/\sigma(I)$	7.0 (0.60)	9.2 (0.4)
Wilson B factor (Å^2)	11.64	11.58
**Refinement**		
Resolution range (Å)	43.29 - 1.30	52.10 - 1.07
No. observations (total/test set)	51931/1682	93126/5022
Completeness (%)	96.40	100
R_{work}/R_{free} (%)	0.14/0.17	0.15/0.17
No. of atoms		
Protein	4056	4034
Ligand/ion	512	565
Waters	90	86
Average *B* all atoms (Å^2)	17.44	17.97
R.m.s. deviations		
Bond lengths (Å)	0.006	0.004
Bond angles (°)	1.301	1.205
**Ramachandran plot**		
outliers (%)	0.0	0.0
allowed (%)	1.41	0.96
favored (%)	98.59	99.04

♭ Numbers in parentheses refer to the highest-resolution shell
@ R_{pim} is the multiplicity weighted, precision-indicating merging R-factor for comparing symmetry-related reflections[26]
+ R_{meas} is the redundancy independent multiplicity weighted R-factor for comparing symmetry related reflections[27]

Supplementary Table 2 - AR3 lattice dimensions, measured in the native claret membrane by atomic force microscopy.

**Protein**	**a (nm)**	**b (nm)**	**α angle (degrees)**
AR3	7.75	7.75	64
Expected values for bacteriorhodopsin[28]	6.2	6.2	65

Supplementary Table 3 – B factors for water molecules within the pentagonal H-bond network between the Schiff Base and Arg92

**Water molecule**	**6GUX - Dark**	**6S6C - Light**
Wat401A	15.83	16.75
Wat401B	15.57	11.78
Wat402	23.77	17.40
Wat406	15.78	16.98

Supplementary Table 4 – Selected distances in Å between sidechains and water molecules within the pentagonal H-bond network between the Schiff Base and Arg92. (Note that the second conformation of Asp95 is not observed in the 6GUX dark-adapted structure and 13-cis retinal is not observed in the 6S6C light-adapted structure.)

**Residue pair**	**Distance / Å**	
	**6GUX - Dark**	**6S6C - Light**
trans Retinal-N to Wat402	2.9	3.3<br>2.8
cis Retinal-N to Wat 402	2.9	
trans Retinal-N to Thr99	3.3	3.3<br>2.9
cis Retinal-N to Thr99	3.3	
Thr99 to Asp95 (1)	2.9	2.9
Thr99 to Asp95 (2)		4.0
Asp95 (1) to Wat402	2.4	2.4
Asp95 (2) to Wat402		2.2
Asp95 (1) to Wat401A	2.8	2.8
Asp95 (2) to Wat401A		2.6
Asp95 (1) to Wat401B	2.6	2.6
Asp95 (2) to Wat401B		2.7
$Wat401_{inner}$ to Wat406	2.6	2.5
$Wat401_{outer}$ to Wat406	3.2	3.1
Wat402 to Asp222	2.9	2.9
Asp222 to Tyr195	2.7	2.7
Arg92 to Wat406	2.8	2.7

Supplementary Table 5 – Selected bond angles within the pentagonal H-bond network between the Schiff Base and Arg92. (Note that the second conformation of Asp95 is not observed in the 6GUX dark-adapted structure.) The internal angles of a regular pentagon are 108° and the H-bond angle in ice is 109.5°.

**Residues**	**6GUX - DA**	**6S6C - LA**
Asp95(1) – Wat402 – Asp222	125.7°	120.2°
Asp95(2) – Wat402 – Asp222		123.6°
Wat402 – Asp222 – Wat406	103.2°	108.6°
Wat401A – Wat406 – Asp222	85.2°	79.0°
Wat401B – Wat406 – Asp222	88.5°	86.8°
Asp95(1) - Wat401A – Wat406	139.8°	146.5°
Asp95(2) - Wat401A – Wat406		145.4°
Asp95(1) - Wat401B – Wat406	121.2°	116.7°
Asp95(2) - Wat401B – Wat406		122.7°

References

1. Nollert, P. Lipidic cubic phases as matrices for membrane protein crystallization. *Methods* **34**, 348–353 (2004).
2. Hussain, R. *et al.* CDApps : integrated software for experimental planning and data processing at beamline B23, Diamond Light Source. Corrigendum . *J. Synchrotron Radiat.* (2015). doi:10.1107/s1600577515007602
3. Whitmore, L. & Wallace, B. A. Protein secondary structure analyses from circular dichroism spectroscopy: Methods and reference databases. *Biopolymers* **89**, 392–400 (2008).
4. Johnson, W. C. Analysing Protein Circular Dichroism Spectra for Accurate Secondary Structures". *Proteins Struct. Funct. Genet.* **35**, 307–312 (1999).
5. Abdul-Gader, A., Miles, A. J. & Wallace, B. A. A reference dataset for the analyses of membrane protein secondary structures and transmembrane residuesusing circular dichroism spectroscopy. *Bioinformatics* **27**, 1630–1636 (2011).
6. Yen, H.-Y. *et al.* Ligand binding to a G protein–coupled receptor captured in a mass spectrometer. *Sci. Adv.* **3**, e1701016 (2017).
7. Fort, K. L. *et al.* Expanding the structural analysis capabilities on an Orbitrap-based mass spectrometer for large macromolecular complexes. *Analyst* **143**, 100–105 (2018).
8. Gault, J. *et al.* High-resolution mass spectrometry of small molecules bound to membrane proteins. *Nat. Methods* **13**, 333–336 (2016).
9. Sobott, F. *et al.* The flight of macromolecular complexes in a mass spectrometer. *Philos. Trans. R. Soc. A Math. Phys. Eng. Sci.* **363**, 379–391 (2005).
10. Gupta, K. *et al.* Identifying key membrane protein lipid interactions using mass spectrometry. *Nat. Protoc.* **13**, 1106–1120 (2018).
11. Jo, S., Kim, T., Iyer, V. G. & Im, W. CHARMM-GUI: A web-based graphical user

interface for CHARMM. *J. Comput. Chem.* (2008). doi:10.1002/jcc.20945
12. Wu, E. L. *et al.* CHARMM-GUI membrane builder toward realistic biological membrane simulations. *Journal of Computational Chemistry* (2014). doi:10.1002/jcc.23702
13. Jo, S., Lim, J. B., Klauda, J. B. & Im, W. CHARMM-GUI membrane builder for mixed bilayers and its application to yeast membranes. *Biophys. J.* (2009). doi:10.1016/j.bpj.2009.04.013
14. Jo, S., Kim, T. & Im, W. Automated builder and database of protein/membrane complexes for molecular dynamics simulations. *PLoS One* (2007). doi:10.1371/journal.pone.0000880
15. Lee, J. *et al.* CHARMM-GUI Membrane Builder for Complex Biological Membrane Simulations with Glycolipids and Lipoglycans. *J. Chem. Theory Comput.* (2019). doi:10.1021/acs.jctc.8b01066
16. Case, D. A. *et al.* Amber 2016. *Univ. California, San Fr.* (2016).
17. Best, R. B. *et al.* Optimization of the additive CHARMM all-atom protein force field targeting improved sampling of the backbone ϕ, ψ and side-chain $\chi 1$ and $\chi 2$ Dihedral Angles. *J. Chem. Theory Comput.* (2012). doi:10.1021/ct300400x
18. Klauda, J. B. *et al.* Update of the CHARMM All-Atom Additive Force Field for Lipids: Validation on Six Lipid Types. *J. Phys. Chem. B* (2010). doi:10.1021/jp101759q
19. Jorgensen, W. L., Chandrasekhar, J., Madura, J. D., Impey, R. W. & Klein, M. L. Comparison of simple potential functions for simulating liquid water. *J. Chem. Phys.* (1983). doi:10.1063/1.445869
20. Beglov, D. & Roux, B. Finite representation of an infinite bulk system: Solvent boundary potential for computer simulations. *J. Chem. Phys.* (1994). doi:10.1063/1.466711
21. Ryckaert, J. P., Ciccotti, G. & Berendsen, H. J. C. Numerical integration of the cartesian equations of motion of a system with constraints: molecular dynamics of n-alkanes. *J. Comput. Phys.* (1977). doi:10.1016/0021-9991(77)90098-5
22. Humphrey, W., Dalke, A. & Schulten, K. VMD: Visual molecular dynamics. *J. Mol. Graph.* (1996). doi:10.1016/0263-7855(96)00018-5
23. PyMOL Molecular Graphics System, Version 2.3.0, Schrödinger, LLC.
24. Wickstrand, C., Dods, R., Royant, A. & Neutze, R. Bacteriorhodopsin: Would the real structural intermediates please stand up? *Biochim. Biophys. Acta - Gen. Subj.* **1850**, 536–553 (2015).
25. Nango, E. *et al.* A three-dimensionalmovie of structural changes in bacteriorhodopsin. *Science (80-.).* **354**, 1552–1557 (2016).
26. Weiss, M. S. & Hilgenfeld, R. On the use of the merging R factor as a quality indicator for X-ray data. *J. Appl. Crystallogr.* (1997). doi:10.1107/S0021889897003907
27. Diederichs, K. & Karplus, P. A. Improved R-factors for diffraction data analysis in macromolecular crystallography. *Nat. Struct. Biol.* (1997). doi:10.1038/nsb0497-269
28. Müller, D. J. *et al.* Atomic force microscopy of native purple membrane. *Biochimica et Biophysica Acta - Bioenergetics* (2000). doi:10.1016/S0005-2728(00)00127-4
