# Supplementary material for: Structures of the archaerhodopsin-3 transporter reveal that disordering of internal water networks underpins receptor sensitization
Source: Nat Commun. 2021 Jan 27;12:629. doi: 10.1038/s41467-020-20596-0 (PMC7840839; doi:10.1038/s41467-020-20596-0)
Supplement: Supplementary file 3 — Reporting Summary [file 41467_2020_20596_MOESM3_ESM.pdf]

# nature research

Corresponding author(s): Anthony Watts

Last updated by author(s): Dec 1, 2020

# Reporting Summary

Nature Research wishes to improve the reproducibility of the work that we publish. This form provides structure for consistency and transparency in reporting. For further information on Nature Research policies, see Authors & Referees and the Editorial Policy Checklist.

## Statistics

For all statistical analyses, confirm that the following items are present in the figure legend, table legend, main text, or Methods section.

| n/a | Confirmed | |
|---|---|---|
| ☐ | ☒ | The exact sample size (*n*) for each experimental group/condition, given as a discrete number and unit of measurement |
| ☐ | ☒ | A statement on whether measurements were taken from distinct samples or whether the same sample was measured repeatedly |
| ☒ | ☐ | The statistical test(s) used AND whether they are one- or two-sided<br>*Only common tests should be described solely by name; describe more complex techniques in the Methods section.* |
| ☒ | ☐ | A description of all covariates tested |
| ☒ | ☐ | A description of any assumptions or corrections, such as tests of normality and adjustment for multiple comparisons |
| ☒ | ☐ | A full description of the statistical parameters including central tendency (e.g. means) or other basic estimates (e.g. regression coefficient) AND variation (e.g. standard deviation) or associated estimates of uncertainty (e.g. confidence intervals) |
| ☒ | ☐ | For null hypothesis testing, the test statistic (e.g. *F*, *t*, *r*) with confidence intervals, effect sizes, degrees of freedom and *P* value noted<br>*Give P values as exact values whenever suitable.* |
| ☒ | ☐ | For Bayesian analysis, information on the choice of priors and Markov chain Monte Carlo settings |
| ☒ | ☐ | For hierarchical and complex designs, identification of the appropriate level for tests and full reporting of outcomes |
| ☒ | ☐ | Estimates of effect sizes (e.g. Cohen's *d*, Pearson's *r*), indicating how they were calculated |

*Our web collection on statistics for biologists contains articles on many of the points above.*

## Software and code

Policy information about availability of computer code

| | |
|---|---|
| Data collection | Xia2 pipeline 0.3.8.0, DIALS 1.10.1, ChemShell 3.7.0 (all previously published and/or commercially available) |
| Data analysis | CCP4 Suite 7.0.066, Phenix 1.18.2, Refmac5, Coot 0.8.9.1, BLEND 0.6.23, Phaser 2.7.17, AIMLESS 0.0.14, CDSSTR, AMBER16, CHARMM36, Prism (version 8), PyMol (version 2.2 and 2.3.0) and ChemDraw Professional 17.1 (all previously published and/or commercially available) |

For manuscripts utilizing custom algorithms or software that are central to the research but not yet described in published literature, software must be made available to editors/reviewers. We strongly encourage code deposition in a community repository (e.g. GitHub). See the Nature Research guidelines for submitting code & software for further information.

## Data

Policy information about availability of data

All manuscripts must include a data availability statement. This statement should provide the following information, where applicable:

- Accession codes, unique identifiers, or web links for publicly available datasets
- A list of figures that have associated raw data
- A description of any restrictions on data availability

Structures and diffraction data have been deposited in the protein databank (https://www.rcsb.org). The accession codes are 6GUX [http://doi.org/10.2210/pdb6GUX/pdb] for dark-adapted AR3 and 6S6C [http://doi.org/10.2210/pdb6S6C/pdb] for light-adapted AR3. Mass spectrometry data are available for download [https://doi.org/10.6084/m9.figshare.13293203.v1]. Other data are available from the corresponding authors upon reasonable request.
In addition to the two crystal structures solved in this study, the paper discusses the following publicly available structures, which have been deposited by other research groups:
1UAZ [http://doi.org/10.2210/pdb1UAZ/pdb] (Archaerhodopsin-1)
3WQJ [http://doi.org/10.2210/pdb3WQJ/pdb] (Archaerhodopsin-2)
5ZIM [http://doi.org/10.2210/pdb5ZIM/pdb] (bacteriorhodopsin)

# Field-specific reporting

Please select the one below that is the best fit for your research. If you are not sure, read the appropriate sections before making your selection.

☒ Life sciences ☐ Behavioural & social sciences ☐ Ecological, evolutionary & environmental sciences

For a reference copy of the document with all sections, see nature.com/documents/nr-reporting-summary-flat.pdf

# Life sciences study design

All studies must disclose on these points even when the disclosure is negative.

| | |
|---|---|
| Sample size | Diffraction datasets were acquired from two separate crystals (51931 reflections in total) for the 6GUX dark-adapted AR3 structure.<br>Diffraction datasets were acquired from 17 separate crystals (93126 reflections in total) for the 6S6C light-adapted AR3 structure.<br>(Further information on the data collection for the crystallography experiments is given in Supplementary Table 1.)<br>Atomic Force Microscopy: Three independent samples were imaged.<br>QM/MM calculation (Fig 3): Each point on the curve is generated from two independent 0.5 ns QM(SCC-DFTB)/MM MD trajectories, initiated from two separated equilibrated starting structures.<br>Mass Spectrometry: Duplicate spectra were acquired from samples prepared independently. |
| Data exclusions | No data were excluded from analysis |
| Replication | Atomic force microscopy: Three independent samples deposited on mica were imaged. The results were found to be reproducible.<br>QM/MM calculation (Fig 3): Each point on the curve is generated from two independent 0.5 ns QM(SCC-DFTB)/MM MD trajectories, initiated from two separated equilibrated starting structures.<br>Mass Spectrometry: Duplicate spectra, acquired from samples prepared independently, showed no significant differences.<br>Crystallography: Crystals were grown reproducibly from three separate protein preparations. In each case X-ray diffraction patterns of similarly high resolution were obtained. |
| Randomization | No experimental group allocation was carried out and so no randomization was performed. |
| Blinding | No experimental group allocation was carried out and so no blinding was performed. |

# Reporting for specific materials, systems and methods

We require information from authors about some types of materials, experimental systems and methods used in many studies. Here, indicate whether each material, system or method listed is relevant to your study. If you are not sure if a list item applies to your research, read the appropriate section before selecting a response.

## Materials & experimental systems

| n/a | Involved in the study |
|---|---|
| ☒ | ☐ Antibodies |
| ☒ | ☐ Eukaryotic cell lines |
| ☒ | ☐ Palaeontology |
| ☒ | ☐ Animals and other organisms |
| ☒ | ☐ Human research participants |
| ☒ | ☐ Clinical data |

## Methods

| n/a | Involved in the study |
|---|---|
| ☒ | ☐ ChIP-seq |
| ☒ | ☐ Flow cytometry |
| ☒ | ☐ MRI-based neuroimaging |
